# Osteoimmuno-Oncology: Therapeutic Opportunities for Targeting Immune Cells in Bone Metastasis

**DOI:** 10.3390/cells10061529

**Published:** 2021-06-17

**Authors:** Tiina E. Kähkönen, Jussi M. Halleen, Jenni Bernoulli

**Affiliations:** 1 OncoBone Ltd., 90810 Oulu, Finland; jussi.halleen@oncobone.com; 2 Institute of Biomedicine, University of Turku, 20500 Turku, Finland; jennibernoulli@gmail.com

**Keywords:** osteoimmuno-oncology, immuno-oncology, osteoimmunology, immunotherapy, bone metastasis

## Abstract

Immunotherapies provide a potential treatment option for currently incurable bone metastases. Bone marrow is an important secondary lymphoid organ with a unique immune contexture. Even at non-disease state immune cells and bone cells interact with each other, bone cells supporting the development of immune cells and immune cells regulating bone turnover. In cancer, tumor cells interfere with this homeostatic process starting from formation of pre-metastatic niche and later supporting growth of bone metastases. In this review, we introduce a novel concept osteoimmuno-oncology (OIO), which refers to interactions between bone, immune and tumor cells in bone metastatic microenvironment. We also discuss therapeutic opportunities of targeting immune cells in bone metastases, and associated efficacy and safety concerns.

## 1. Immuno-Oncology and Bone

Immunotherapies have become a groundbreaking therapy for many cancers [1]. The number of new immunotherapies in different phases of development has increased considerably over the past decade and continues to increase constantly [2]. Throughout the development of immunotherapies, we have learned that some tumors respond better than others [3]. This has led to classification of tumors into the ‘hot’ or ‘inflamed’ type when they have a high number of immune cells in the tumor microenvironment (TME), typically resulting in a better response rate to immunotherapies [4]. Not so well responding tumors are classified as ‘cold’ or ‘immune-desert’ type with low number of infiltrated immune cells [5]. Additionally, other properties, such as high tumor mutational burden (TMB) that affects the amount of neoantigens in tumors, have been identified as factors that make tumors more responsive to immunotherapies [6]. Even though these classifications have provided some assistance in guiding immunotherapy development, a better understanding of TME and the development of novel predictive biomarkers would help to identify patients that respond to immunotherapies [7,8]. The current consensus is that immunotherapies, as stand-alone therapies, can provide durable responses in some patients, but in most cases, they need to be combined with other therapeutic modalities for better overall response rate [9].

### 1.1. Bone Microenvironment

Many decades have been spent on studying and understanding unique aspects of TME [10,11,12]. The uniqueness of the TME becomes especially challenging when cancer forms metastasis to distant organs that each have unique properties. This uniqueness is gained during complicated events of overlapping processes in the metastatic progression [13] that start by tumor cells changing their properties through an epithelial-to-mesenchymal transition (EMT) [14,15], allowing them to leave the primary tumor (intravasation [16]), entera new TME (extravasation [17]), and modulate the new TME to allow the growth of secondary tumors called metastases. It is essential to understand that the tumor that was once growing in the primary site changes during the metastatic process and typically becomes resistant to standard-of-care therapies due to the influence of the metastatic TME [18,19,20].

Bone is an especially unique metastatic TME. Bone metastases are very common and cause high mortality in some of the most common cancers, such as breast, prostate, and lung cancer [21,22,23]. Bone metastasis can be divided into osteolytic, osteosclerotic (osteoblastic), or mixed lesions [24]. Osteolytic bone metastases induce rapid bone resorption, and they are common in breast and lung cancer. In contrast, osteoblastic bone metastases induce formation of pathologic new bone, and they are common in prostate cancer. Common for both types of bone metastases is an increased risk of fractures, bone pain, and decreased quality of life [25]. Better understanding and consideration of the metastatic TME in bone would allow to develop novel therapies that would be effective in bone metastases, which to date remain incurable. One compelling approach in this manner are immunotherapies. Bone is an important secondary immune organ and immune cells have multiple interactions with bone marrow stromal cells, and they regulate each other’s function and activity [26,27]. Tumor cells further modulate the immunological content in the bone metastatic microenvironment mostly by making it immunosuppressive, leading to a low response rate to different cancer therapies [28].

**Figure 1:**
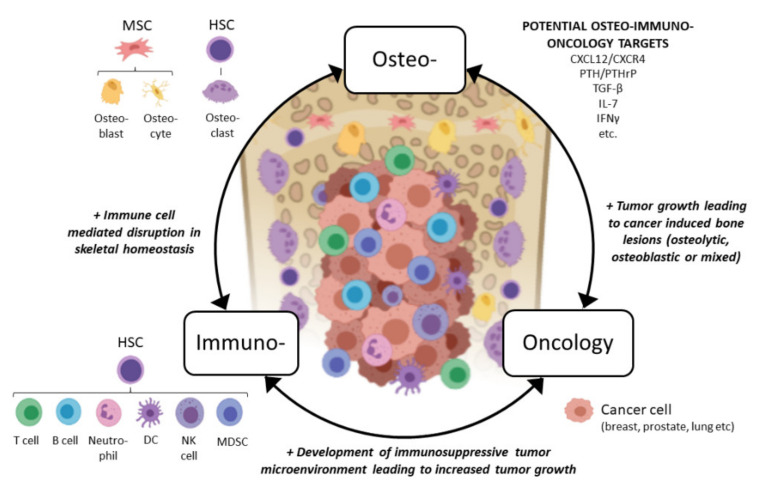
Concept of osteoimmuno-oncology and emerging potential targets for patients with bone metastases. Abbreviations: MSC: mesenchymal stem cell, HSC: hematopoietic stem cell, DC: dendritic cell, NK: natural killer, MDSC: myeloid-derived suppressor cell, CXCL12: CXC chemokine ligand 12, CXCR4: CXC chemokine receptor type 4, PTH: parathyroidhormone, PTHrP: PTH-related peptide, TGF-β: transforming growth factor beta, IL-7: interleukin 7, IFN-γ: interferon gamma. Figure created with BioRender.com.

In light of the above, we would like to propose a novel osteoimmuno-oncology (OIO) approach when developing immuno-oncology compounds for treating bone metastases (Figure 1). This means that all three aspects, bone cell interactions with immune cells (osteoimmunology), immune cell interactions with tumor cells (immuno-oncology), and tumor cell interactions with bone cells, would be considered when developing more effective compounds for patients with the currently incurable bone metastases. In the next sections, we discuss interactions between bone marrow immune cells and bone cells, a field commonly overlooked by oncology researchers, and how the immune cells affect the growth of bone metastases from an OIO aspect. We also discuss the immunological effects of emerging therapies used in bone metastatic patients and future remarks in this field.

### 1.2. Bone Marrow Immune Cells

Bone marrow is an important secondary lymphoid organ [26]. About 25% of bone marrow immune cells are myeloid cells, about 10% are lymphocytes, and about 5% are dendritic cells, antigen-producing plasma cells, and natural killer cells [26]. Bone marrow can support T cell development in the absence of thymus [29], B cells are matured in bone marrow and leave the marrow in response to erythropoietin [30,31], and antigen-producing plasma cells in the bone marrow can cause immune reactions [32].

Bone marrow-residing cells, such as leukocytes, hematopoietic stem cells (HSCs), and stromal cells, secrete cytokines that are important for the development and function of different immune cells. For example, interleukin (IL) -7 is involved in transition of bone marrow progenitor cells to thymus [33] and bone marrow stromal cell-secreted IL-7 is essential for early B cell development [34,35]. IL-15, secreted by bone marrow stromal cells [36], can inhibit IL-7 receptor α-chain, which is needed for T cell differentiation [37]. IL-15 can also stimulate both myeloid and lymphoid cell proliferation and, together with IL-21 delay, apoptosis of bone marrow cells [38]. Chemokines are regulators of the innate immune system and the largest chemokine families are CC chemokines and CXC chemokines that act by binding to corresponding receptors [39]. Chemokine CXCL-12 is involved in colonization of CXCR4 expressing cells, such as HSCs and lymphocytes, to the bone marrow [26]. CXCR4/CXCL12 signaling further regulates memory T cell, Treg, and neutrophil trafficking to bone marrow [40,41]. Adhesion molecules VCAM-1 and E-selectin are necessary for recruitment of hematopoietic progenitors, lymphocytes and developing neutrophils to the bone marrow and VCAM-1/VLA-4 are needed for the release of mature neutrophils from the bone marrow [42]. Furthermore, VCAM-1 is needed for the maturation of B cells and plasma cells [43] and migration of B cells, CD4+ and CD8+ T cells to the bone marrow, and E-selectin is needed for migration of CD8+ memory T cells to the bone marrow [26].

T and B cells have a role in bone homeostasis by directly interacting with bone cells, which will be discussed in the following section.

### 1.3. Osteoimmunology

Osteoimmunology is defined as an interdisciplinary research field focusing to understand the diverse interplay between the immune and skeletal system [44]. This interplay is a dynamic bidirectional process both in healthy and diseased states. Osteoimmunological studies are increasingly carried out in skeletal diseases, including bone metastases [45]. In the following sections, we discuss the cell types involved in osteoimmunology, including the differentiation of mesenchymal stem cells (MSCs) to bone-forming osteoblasts and osteoblast regulation of HSCs that differentiate into bone-resorbing osteoclasts and immune cells.

Multipotent MSCs can differentiate into bone-forming osteoblasts that later differentiate into osteocytes [46]. The primary function of the osteoblast is to secrete mineralized extracellular matrix, but it also has an important function in regulating HSC niches [47]. Osteoblasts produce cytokines such as granulocyte colony-stimulating factor (G-CSF), IL-1, IL-6, IL-7, thrombopoietin, angiopoietin 1 and the chemokine CXCL12 that help to maintain HSC niches [27]. Osteoblasts also express many types of adhesion molecules, such as vascular cell adhesion molecule 1 (VCAM-1) [48] and intercellular adhesion molecule 1 (ICAM-1), together with CD44 [49], which might facilitate interactions within the HSC niche.

Osteoblast interactions with HSCs are important because HSCs differentiate into bone-resorbing osteoclasts. Osteoblasts and osteoclasts regulate the homeostatic balance between bone formation and resorption. Furthermore, HSCs are the progenitors of all immune cells, such as T and B cells [50]. Signals coming from mature osteoblasts are important for lymphoid progenitor cells and generation of T and B cells. Loss of osteoblast-derived signals, osteocalcin and osterix, decrease the number of T cell progenitors [27]. Additionally, T cell maturation is decreased when osteoblasts do not secrete Delta-like protein 4, whose actions are mediated by Notch signaling [27]. On the contrary, activated Th17 cells increase osteoblast differentiation via IFN-γ [2] and TGF-β [51] and Foxp3+ regulatory T cells increase osteoblast differentiation when stimulated with dehydroepiandrosterone (DHEA) [52]. Bone marrow T cells are involved in G-CSF-induced activation of osteoclasts [53]. Osteoblasts also affect B cell development [54]. In conditional knockout mice, ablation of osteoblasts blocks B cells differentiation from HSCs [27]. Sclerostin knockout mice have increased B cell apoptosis resulting in complete loss of these cells in bone marrow [55], which is due to missing cell-cell contacts with VCAM-1 expressing osteoblasts and local secretion of IL-7 and CXCL12 [27]. IL-7 and IL-12 can further decrease osteoblast differentiation via osteoclast modulation of the bone marrow microenvironment [56]. Additionally, parathyroid hormone (PTH) receptor-mediated signaling in osteoblasts is necessary for B cell differentiation via IL-7 and in their mobilization via VCAM-1 [57]. Furthermore, disabling the signaling of the PTH-related peptide (PTHrP) receptor decreases the number of B cell precursors in the bone marrow [27]. In addition, osteocytes are involved in the maintenance of myeloid cells [27].

As mentioned previously, immune cells regulate the function of bone cells. T cells regulate bone turnover by promoting osteoclastogenesis and bone loss via RANKL, but they also have an opposite effect on osteoclastogenesis through IFN-gamma production [58]. Deletion of RANKL from T cells increases trabecular bone formation [59]. CD4+ T cells increase osteoclastogenesis by affecting RANKL/OPG ratio [60] and RANKL secreted by B cells increases osteoclastogenesis [61]. Additionally, Tregs [62] and NKT [63] mediate bone loss via RANKL. B cells are important for the development of osteoblasts and osteoclasts [64]. Deletion of RANKL from B cells alters the number of these cells in the bone marrow without preventing their maturation, indicating an autocrine function of RANKL in B cell maturation. Moreover, RANKL produced by osteocytes and increase in B cells are both needed for bone loss at least in estrogen-deficient conditions [65]. Many other factors, such as IL-7, CXCL12 and PTHrP receptor, support B cell development and, thus, regulate bone loss, and many cytokines secreted by immune cells affect the skeletal system [66].

Physiological bone turnover is disturbed in many diseases such as bone metastases. As was discussed in previous paragraphs, immune cells have a role in the maintenance of bone homeostasis. It is widely acknowledged that the number of immune cells change in cancer metastasis, but their role in the regulation of bone metastasis growth is not well-described. In the following sections, we discuss the presence and actions of immune cells in the bone metastatic microenvironment from the OIO aspect.

## 2. Immune Cells in Bone Metastasis

Bone metastases are currently incurable. Current treatments are palliative at best, including chemotherapy, radiotherapy, targeted therapies, and anti-resorptive therapies that aim to slow down bone destruction induced by cancer cells [67]. No real evidence exists that patients with bone metastases would benefit from immune cell-targeted therapies. Some immunotherapies have provided survival benefit for advanced cancer patients, including ipilimumab in metastatic melanoma and sipuleucel-T, an immunotherapy manufactured from a patient’s peripheral blood mononuclear cells in advanced prostate cancer [68]. New research has provided similar findings, for example for pembrolizumab in bone metastatic prostate cancer [69]. This highlights that better understanding of immune cells in bone metastasis is needed in order to develop effective therapies.

Currently, immune cells, such as T cells (CD4+, CD8+ and Tregs), MDSCs, macrophages, neutrophils, and NK cells, are targeted for treating bone metastases [70]. However, most bone metastases have typically low immunogenicity compared to the primary tumor, and therefore, they may respond poorly to immunotherapies. This is currently not well acknowledged, which could be due to a lack of understanding the role of the bone microenvironment and osteo-immuno-oncological interactions in controlling disease growth and resistance to therapies, accompanied with lack of knowledge of using appropriate metastasis models in preclinical research and the low availability of biopsies from bone metastases. It is important to understand how current treatments affect the immune landscape in bone metastatic microenvironment, which is shortly discussed in the last sections of this review.

In the next sections, we will discuss the different immune cell subtypes and their effects on bone metastases, which are also summarized in Table 1 below.

### 2.1. T Cells

T cell targeting therapies, including checkpoint inhibitors for PD-1, PD-L1, and CTLA-4, were the first immunotherapies approved for treatment of cancer, and there are currently many other therapies emerging [118].

T cells in bone metastatic microenvironment comprise CD8+ cytotoxic T cells, CD4+ helper T cells, regulatory T cells (Tregs), and natural killer T (NKT) cells. Interactions between T cells and tumor cells in the bone microenvironment are considered one important reason for metastatic relapse [119]. Early in the metastatic process, T cells are involved in the formation of pre-metastatic niche in tumor cell arrival in bone. In the pre-metastatic niche, T cells induce osteolysis by increasing expression of pro-osteoclastic cytokines including RANKL [120]. RANKL seems to be a major regulator in the premetastatic niche, since deletion of RANKL from tumor-specific T cells dismisses the formation of metastases, highlighting the importance of this interaction [120]. Cancer cells in bone metastases produce factors such as PTHrP, IL-7, and IL-8 that can activate T cells [71,121]. In contrast, T cells affect bone cells, resulting in increased cancer-induced osteolysis [71] and the release of factors such as TGF-β that can suppress proliferation and function of T cells and their anti-tumor effects by increasing conversion of CD4+ T cells to Tregs, creating immunosuppressive microenvironment and increasing the formation of metastases [71,72,73]. Furthermore, the transfer of activated T cells to breast cancer patients with pre-existing tumor-reactive bone marrow T cells causes anti-tumor effects especially in patients with bone metastases [122]. T cells can also regulate tumor growth in bone independently from interactions with bone cells [68]. For example, RANK/RANKL interactions with CD4+ T cells and breast cancer cells promotes invasion and dissemination of tumor cells and the formation of bone metastases [73].

CD4+ and CD8+ T cells have anti-tumor activity in both primary and metastatic tumors [68]. CD4+ T cells are important in orchestrating immune responses in cancer and in priming and survival of CD8+ T cells. Additionally, CD4+ T cells that encounter mutated genes such as fibronectin are involved in tumor metastasis [74]. CD8+ T cells are mostly responsible for immune-mediated tumor cell death.

Clinical evidence shows that in ovarian cancer patients, together with increased PD-L1, a high number of intratumoral CD8+ T cells correlates with advanced and metastatic stages. This is further supported by co-culture experiments with ovarian cancer cells and CD8+ T cells, where increased migration, invasion, and expression of metastasis-associated genes such as MMP-9, VEGF, IL-8 and IL-10, and PD-L1 are observed [74]. In fact, anti-MMP-9 treatment of mice with breast cancer increases immune signature pathways responsible for T cell activation [123]. Interferon regulatory factor 7, together with NK and CD8+ cells, is associated with longer metastasis-free survival in breast cancer [75]. A study in a spontaneous melanoma mouse model shows that CD8+ T cells have no effect on primary tumor formation, but they control disease progression and formation of metastases [76]. In prostate cancer, loss of PTEN in bone metastasis is associated with a low number of CD8+ T cells [77]. This points towards the initiative to treat patients with early metastatic disease compared to late-stage disease patients. At a late stage, the bone metastatic microenvironment may have a low number of or lacking CD8+ T cells. An alternative option is to develop therapies that increase the number of CD8+ T cells in the bone metastatic microenvironment that could be combined with for example checkpoint inhibitors that affect through CD8+ T cells for better anti-tumor response.

CD4+ T cell subsets, Th17 cells and Tregs increase the growth of bone metastases by increasing RANKL-mediated osteoclastogenesis [78]. Th17 cells have a role in bone marrow immunity irrespective of tumor presence. The number of Th17 cells is increased via IL-1, IL-6, and TGF-β in multiple myeloma bone disease [26]. RANKL-expressing Th17 cells can later differentiate into Tregs by exposure to TGF-β and aryl hydrocarbon receptor (AhR) in the bone microenvironment [78]. A high number of bone marrow Tregs is associated with the development of metastatic disease [124]. Tregs are immunosuppressive cells whose number is increased in almost all cancer patients, and their presence correlates with poor prognosis [26,78]. In ovarian cancer, a high number of Tregs is associated with advanced stage but not survival [79]. The number of Treg increases in bone metastatic prostate cancer patients and Tregs can also regulate cancer-induced bone resorption [78]. Treg trafficking to bone marrow is mediated by CXCR4/CXCL12 interactions, and bone marrow is the preferential site for migration, retainment, and function of Tregs. Furthermore, RANKL expressing Tregs can promote tumor cells homing to the bone marrow [78]. COX2 overexpression leads to increased number of Tregs in primary breast cancer and development of bone metastases [80].

Bone marrow NKTs can regulate immune responses and reject tumor cells [26]. In metastatic breast cancer cells, expression of the MHC class I-like molecule CD1b is downregulated, disabling antigen presentation to NKT cells, which leads to decreased anti-tumor responses and the formation of metastases [81].

### 2.2. NK Cells

Natural Killer (NK) cells are important mediators in tumor immune surveillance, but their role both in bone physiology and bone metastasis is poorly understood [125]. NK cells recognize antigen-specific receptors such as NKG2D and DNAM1 expressed on tumor cells [126] or tumor cells that have downregulated expression of MHC molecules [78]. The dysfunction of NK cells has been observed in many cancers, including prostate cancer, which may be caused by production of reactive oxygen species (ROS) mainly by tumor cells [78].

The deletion of NK cells induces tumor growth and the formation of metastases [78]. In an experimental prostate cancer model, metastasis progression was associated with loss of peripheral NK cells [82]. IFN-γ signaling seems to be critical for pro-metastatic effects of NK cells. For example, NK cells suppress metastasis via IFN signaling in a preclinical breast cancer model [75]. Injection of murine IL-12 activates NK cells and decreases metastasis, suggesting that IFN-γ is also needed in NK cell -mediated metastasis via IL-12 [83]. Anti-metastatic potential of NK cells has also been shown in cancer models, where metastases were diminished by the deletion of NK cells, treatment with IFN-γ neutralizing antibody, and in CD39-deficient models [127]. In an experimental prostate cancer model, overexpression of IRF7 results in increased bone metastases via IFN-beta and increased activity of NK cells [128]. NK cells prevent formation of metastasis in a humanized mouse model, but metastases are formed when NK cell-mediated tumor differentiation is blocked with IFN-y or TNF-alpha antibodies [84]. Furthermore, studies with syngeneic breast cancer models in IFN knockout mice have shown that IFN signaling to hematopoietic system determines metastasis-free survival and responsiveness to circulating NK cells [85].

There are also other factors that affect NK cell-mediated immune responses. Core2 β-1,6-N-acetylglucosaminyltransferase (C2GnT) expressed on tumor cells disturbs NK-mediated immune responses and cancer cell apoptosis in bone metastasis [78]. The activity of TAM tyrosine kinase receptors regulates NK cells and the formation of metastases in a breast cancer model [78]. IL-17A is a major regulator of NK cells, and studies in IL-17A knockout mice shows suppression of metastases caused by increased maturation and activity of NK cells [86]. IL-28R is important for NK cell function and IL-28R knockout mice have an elevated number of metastases [87]. Studies in NK cell-depleted mice show that treatment with PTHrP neutralizing antibody [129], the bisphosphonate minodronate [88], the follistatin inhibitor activin [130], the VEGF antibody bevacizumab, and the bisphosphonate zoledronic acid [131] inhibit bone metastasis from lung cancer cells. JAK/STAT pathway is active in breast cancer patients and in preclinical models, but inhibition of JAK signaling increases metastatic tumor burden due to the impairment of NK-mediated anti-tumor activity [89].

As discussed previously, TGF-β is important both for bone and immune cells. In patients, high TGF-β serum levels correlate with NK cell -mediated immunosuppression and poor clinical outcome [68]. Inhibition of TGF-β signaling increases the anti-tumor activity of NK cells and prevents the formation of bone metastases in an experimental breast cancer model [90], but the direct effects between TGF-β and NK cells remain to be studied.

### 2.3. MDSCs

Myeloid-derived suppressor cells (MDSCs) are a heterogeneous population of cells generated in the bone marrow from immature myeloid cells. Normally, immature myeloid cells differentiate into mature myeloid cells including macrophages and dendritic cells. With cancer, this normal differentiation is disturbed by cancer cell-produced immunosuppressive factors, which causes immature myeloid cells to proliferate, differentiate, and become active MDSCs [78,86]. Immunosuppressive factors responsible for this include, for example, arginase I, inducible nitric oxidase synthase (iNOS), TNF-α, and TGF-β [68,78]. MDSCs accumulate to almost all tumor types and create an immunosuppressive tumor microenvironment by secreting factors such as arginase I [132], inducible nitric oxidate synthase (iNOS) [133], TGF-β and IL-10 [134], as well as other immunosuppressive factors [135]. MDSCs suppress tumor-directed immune responses by suppressing T cell proliferation, inducing T cell apoptosis, activating Tregs for immunosuppression, and decreasing the activity of NK cells. Cytokines activating metastasis that are linked to MDCS include CCL5, CCL15 [136], and pro-metastasis proteins such as MMPs and chemoattractants [91]. Furthermore, monocytic MDSCs are associated with a trend towards decreased survival in patients with liver and bone metastases [92].

MDSCs can further direct metastasis to the bone. Increased number of MDSCs is associated with increased bone metastasis in a breast cancer animal model [78]. Interestingly, the number of MDSCs is increased in the metastatic location in a breast cancer model, but after the removal of the tumor, the number of other than granulocytic MDCSs decreases in the metastatic location [93]. Furthermore, the prevention of tumor accumulation of granulocytic MDSCs leads to anti-metastatic effects in a breast cancer model [94]. High expression of the TGFβ growth factor family member BMP4 results in inhibition of metastasis [95]. Inhibition of CXCR4 decreases the number of MDSCs and Tregs, resulting in the progression of bone metastases [127]. The treatment of mice with a conjugate of paclitaxel and a muramyl dipeptide analogue decreases tumor growth and metastasis in breast and lung cancer models by suppression of MDSCs [137]. Furthermore, dexamethasone treatment decreases myeloid MDSCs and results in decreased tumor growth [97].

MDSCs have also an important function in bone, and the number of MDSCs in bone metastasis in higher compared to primary tumors or other metastatic locations [73]. In a breast cancer mouse model, injection of MDSCs induces reportion in mice with bone metastases but not in healthy mice [138]. Bone marrow MDSCs can differentiate into osteoclasts [98] and contribute to cancer-induced bone destruction [138], therefore contributing to the vicious cycle of bone metastasis. MDSCs also contribute to the production of IL-17 in bone, which can increase osteoclastogenesis via RANKL [68]. MDSCs express CCR2 and CCL2, which are important for osteoclastogenesis, and cancer cells secrete CCL2, CCL5, and osteopontin, which enhance osteoclast function [73]. Additionally, MDSCs enhance the growth of bone metastases independently of their ability to differentiate into functional osteoclasts [73].

MDSCs provide an interesting target for anti-cancer therapy, and currently, there are many therapeutics that affect MDSCs [139]. From a bone metastasis perspective, these include, for example, zoledronic acid, which decreases the number of circulating MDSCs by inducing their apoptosis, chemokine receptor antagonists of CCR2, CXCR2, and CXCR4, and chemokine inhibitors of CCL2, CXCL5, and CXCL12, which inhibit MDSC accumulation into tumor [78].

### 2.4. Macrophages

In tumor microenvironments, macrophages can become tumor-associated macrophages (TAMs) [140]. There are two different subsets of macrophages that polarize when stimulated by different cytokines in their tissue microenvironment, pro-inflammatory M1/M1-like and anti-inflammatory M2/M2-like macrophages [78,141]. M1 macrophages and secrete proinflammatory cytokines such as IL-1, IL-6, IL-12, IL-23, and IFN-γ that can activate T and NK cells [78]. M2/M2-like macrophage differentiation is influenced by cytokines such as TGF-β and IL-10 [141], and they secrete anti-inflammatory cytokines such as IL-10, TGF-β, CCL17, CCL18, CCL22, and CCL24, and promote tumorigenesis and later development of metastases [141].

TAMs are activated by IL-10 and TGF-β, secrete high levels of cytokines that decrease the activation of T cells, and participate in tumor progression and the formation of metastases. For example, BMP4-containing conditioned medium collected from bladder cancer cells increases M2 macrophage differentiation [99], IL-6 and GM-CSF produced by cancer-associated fibroblasts induce M2 macrophage differentiation [142], and caspase-1 promotes TAM differentiation by cleaving PPARy, which then interacts with medium-chain acyl-CoA dehydrogenase (MCAD) [100]. Furthermore, inhibition of caspase-1 or deletion of MCAD decreases tumor growth.

A high number of TAMs correlates with poor clinical outcome in patients in many cancers [78]. However, a study in osteosarcoma patients without metastases shows increased infiltration of both M1 and M2 macrophages with overall improved survival and reduced metastasis [101], which might indicate that the balance between M1 and M2 macrophages is important in this aspect. TAMs can be targeted in cancer by depletion, reprogramming, or molecular targeting [78].

TAMs potentiate bone metastasis [143], which has been shown for example in lung cancer, where macrophages migrate towards CXCL14 expressing bone-seeking cells more efficiently than towards parental lung cancer cells [102], and deletion of macrophages suppresses bone metastasis of lung cancer [103]. In a prostate cancer bone metastasis model, M2 macrophages are observed and treatment with trabectedin reduces M2 macrophages and growth of bone metastases [104]. A high expression of WNT5A is associated with high levels of CCL2, and BMP6 is commonly observed in prostate cancer cells growing in a bone microenvironment [144]. Furthermore, deletion of macrophages decreases the castration resistance of prostate cancer cells [144]. CD169+ macrophages support tumor growth and metastasis in breast cancer [105], and when they are deleted, the number of CD8+ T cells increases but bone loss remains the same [105]. Deletion of mTOR signaling complex 2 (mTORC2) from monocytes and their injection to mice in a breast cancer model results in prevention of the monocytes to differentiate to M2 macrophages and formation of distant, including bone, metastases [106]. Bone marrow-derived macrophages are recruited by tumor chemokines such as CCL2 and CSF-1 or their receptors and promote metastasis to bones. CSF-1/CSF-R1 targeted therapy provides anti-metastatic effects [78], and CCL2 and CCLR2 signaling causes accumulation of TAMs into tumors, which reduces metastasis in animal models [78]. However, CCL2-targeted therapy increases metastasis via increased angiogenesis in a breast cancer model [78].

Macrophages also have effects on cancer-induced formation of new bone. In prostate cancer bone metastasis specimens, CD68+ macrophages are consistently located in the tumor, compared to variable distribution of lymphocytes, and osteal macrophages are located near pathologic new bone [107]. Deletion of CD169+ macrophages inhibits the formation of pathologic new bone in a prostate cancer model, and deletion of other macrophages than CD169+ increases tumor growth [107]. Furthermore, CD137+ cells promote the migration of macrophages and monocytes to tumor and further promote their differentiation into osteoclasts, increasing cancer-induced bone destruction in a spontaneous breast cancer model [145].

### 2.5. Dendritic Cells

Dendritic cells (DCs) are antigen-presenting cells that regulate T cell responses by increasing their proliferation and activation [78]. DCs are divided into monocytic DCs (mDCs) and plasmacytoid DCs (pDCs), and pDCs are mainly studied in immuno-oncology [68].

Circulating DCs migrate to bone marrow and engage with VCAM-2 and selectin-expressing cells. DCs that are infiltrated to bone metastases derived from low-immunogenic tumors are typically immature and lack antigen-presenting capacity [68]. Furthermore, prostate cancer bone metastatic microenvironment inhibits DC maturation [68]. RM-1 prostate cancer cells growing in bone marrow decrease DC generation and their capability to increase T cell proliferation [108]. This might be partially due to the expression of PD-L1 in bone marrow DCs and PD-L1-mediated deactivation of CD8+ T cells [68]. In breast cancer, DCs purified from cancer patients have a decreased ability to stimulate T cells [78], and tumor-infiltrating DCs suppress the function of CD8+ T cells via TGF-β, NO, IL-10, VEGF, and arginase I [78]. The number of pDCs is increased in a breast cancer bone metastasis model [78]. DCs increase metastasis and decrease survival in Batf3-deficient mice, but they have no effect on primary tumors [83]. The increase in metastasis via Batf3 requires NK cells and IFN-γ, but not CD4+ or CD8+ T cells. DCs can also recruit immunosuppressive Treg and MDSCs into tumor [78].

Tumors with high TGF-β expression have less DCs [109]. TGF-β inhibitor induces maturation of DCs with increased production of IL-12, and can potentially activate DCs into NK cells [110]. Inhibition of CD115/CSF-1 decreases osteoclast differentiation and prevents monocyte differentiation to M2 macrophages and instead promotes their differentiation into DCs [146].

Sipuleucel-T is a DC-targeted therapy for castration-resistant prostate cancer (CRPC) patients that provides survival benefit [68]. DC-based therapeutic approaches are related to vaccines, for example in breast, prostate, and lung cancer, and these vaccines can also prevent the formation of metastases [78]. DC vaccines also require NK cells and CD8+ cells to function properly [147]. The prevention of metastases is seen with mature DCs and 5-FU treatment [78]. Combination of stontium-89 and DC vaccination therapy provides a good response in patients with bone metastases [111]. Activation of DC with tumor-lysates and combination with anti-CTLA-4 treatment increases the number of CD8+ T cells, decreases the number of Tregs, and inhibits metastatic growth [112]. However, infiltration of DCs into tumors after injections may be problematic [148].

### 2.6. Neutrophils

Mature neutrophils reside in bone marrow and bone marrow regulates their homeostasis via the CXCR4/CXCL12 signaling pathway [78]. Different chemokines regulate the anti- or pro-tumoral effects of neutrophils [113], and, for example, the inactivation of atypical chemokine receptors (ACKRs) results in the release of neutrophils from bone marrow that have anti-metastatic activity [114]. Neutrophils regulate formation of pre-metastatic niche [115]. Interestingly, lung carcinoma increases bone stromal activity in the pre-metastatic niche by affecting neutrophils that express high levels of SiglecF through osteocalcin-expressing osteoblastic cells [149]. Neutrophils also have a role in early metastasis development, as a high number of migrating neutrophils with immunosuppressive properties are observed in tumor-bearing mice [116]. In tumor microenvironments, neutrophils can become tumor-associated neutrophils (TANs) that can be either tumor-inhibiting (N1) or tumor-promoting (N2). TGF-β is an important mediator in differentiating between N1 and N2 neutrophils [78]. N2 neutrophils promote angiogenesis, dissemination of tumor cells, and metastasis formation, including metastasis to bones [78]. N2 neutrophils can also release CXCR4, VEGF, and MMP9, which all have well-defined roles in metastasis.

The neutrophil-to-lymphocyte ratio is an independent prognostic marker in patients with bone metastases [150,151]. The neutrophil-to-lymphocyte ratio correlates with PSA, being high in patients with bone metastatic prostate cancer [127]. However, the neutrophil-to-lymphocyte ratio seems to be typically higher in patients with soft tissue metastases [152]. Furthermore, prostate cancer stimulates recruitment of neutrophils that infiltrate heavily to bone metastatic lesions, and at early stages, neutrophils induce prostate cancer cell apoptosis, but at later stages, they fail to execute cytotoxic responses [117].

## 3. Therapeutic Approaches

Patients with bone metastases are at high risk for bone-related complications, such as pain and fractures, and preservation of bone health is, therefore, important for these patients. Bone-modifying agents that are used as supportive treatment to maintain bone mass from excess remodeling in patients with osteolytic bone metastases include bisphosphonates and anti-RANKL antibody [153]. Radium-223 dichloride is indicated for use in the management of CRPC patients with symptomatic bone metastases to prevent excess bone formation in osteoblastic bone metastases. As per osteo-immuno-oncological interactions in bone metastases, it is not surprising that current and emerging bone-targeted therapies also have immunological effects that can partially cause anti-tumor effects.

There are also other potential therapeutic targets for bone metastasis. The osteoclast is the major cell type responsible for the development of bone metastases and cancer-induced bone changes, and as described above, targeting osteoclast function has provided clinical benefit for patients with bone metastases. Understanding osteoclast signaling in bone metastasis could help to identify new targets for drug discovery [153], and there are currently drug candidates in different phases of development that are targeting osteoclasts, such as SRC, DKK-1, and Sclerostin-targeting compounds [154]. Additionally, importantly, interactions with other cell types in the bone metastatic microenvironment could potentially provide new treatment options for bone metastases [155].

### 3.1. Bisphosphonates

Bisphosphonates are anti-resorptive agents widely used for the treatment of bone metastases in various cancer types [71,156,157]. There are three generations of bisphosphonates, of which zoledronic acid is considered the most effective in terms of preventing the formation of bone metastases in an adjuvant setting, but also in reducing morbidity from bone metastases. The primary effect of zoledronic acid is to induce osteoclast apoptosis, and thus, prevent cancer-induced osteolysis [158,159].

Zoledronic acid has many immunological effects [160] through the modulation of gamma delta T cell activity in many cancers, such as multiple myeloma [161]. It has been reported to decrease M2 macrophage differentiation [162,163] and their phagocytic activity [164], and via decreasing M2 activity, increases T cell-mediated cytotoxic effects [165]. Zoledronic acid also affects stromal cells such as cancer-associated fibroblasts (CAFs) that affect M2 polarization and decrease prostate tumor growth and metastasis [166]. Zoledronic acid decreases proliferation and immunosuppressive effects of Tregs [167], and downregulates expression of CTLA-4 and PD-1 on Tregs [168]. In a triple-negative breast cancer (TNBC) mouse model, the combination treatment of anti-PD-1 and zoledronic acid is more efficacious than the single treatments [169]. A zoledronic acid-induced decrease in Tregs is associated with increased proliferation of T and NK cells in metastatic patients [170]. Zoledronic acid treatment may also have metastasis-suppressive effects, as in mice treated with zoledronic acid, the bone marrow is changed to metastasis-suppressive by modulating myeloid/osteoclast progenitor cells via G-CSF [171]. The transfer of gamma delta T cells has been considered as one treatment option for cancer. The transfer of gamma delta T cells in zoledronic acid-treated mice decreases tumor growth and tumor-induced osteolysis in a breast cancer model [172], and promising findings have also been demonstrated in clinical trials [173,174]. Another treatment option has been to combine zoledronic acid with IL-2 therapy, which has now been tested in metastatic renal cell carcinoma [175], breast cancer [176], hormone refractory prostate cancer [177], and in patients with metastatic cancer [178].

### 3.2. RANK-Ligand Inhibition

Denosumab is an anti-RANKL antibody that inhibits cancer-induced bone resorption in bone metastasis [179,180]. Denosumab has also immunological effects through T cells, which creates a potential for combination treatment with immunotherapies [181,182]. For example, in melanoma bone metastatic patients treated with anti-CTLA-4 and denosumab [183] and with anti-PD-1 and denosumab [184], promising efficacy is observed [185]. Combining anti-CTLA-4 and denosumab is further supported by preclinical findings from a metastasis model that showed improved efficacy with increased T cell infiltration [186]. Furthermore, RANKL inhibition increases the responsiveness to immunotherapies, which would be especially valuable in the case of bone metastases [187].

### 3.3. Radiopharmaceuticals

Radium-223 dichloride is approved for the treatment of CRPC patients with bone metastases [188]. As a calcium mimetic, radium-223 dichloride accumulates in bone, especially in high turnover states such as in cancer bone metastasis [188].

The recently published results of a phase 2 study evaluated potential increase in immunogenicity due to radium-223 and pembrolizumab treatment in metastatic CRCP patients with bone metastases [189]. In paired bone biopsy samples, no change in CD4+ or CD8+ cells is observed, and about 10% of the patients have a PSA response. Interestingly, radium-223 dichloride treatment of patients combined with prednisone decreases PD-1 expressing effector memory CD8+ T cells in peripheral blood [190]. This immunosuppression can, however, be due to concomitant dexamethasone treatment [191]. Radium-223 has also been studied in combination with sipuleucel-T. Interim results of a phase 2 combination study suggest synergistic effects, but further studies are warranted [192]. In relation to radium-223, monitoring and maintaining bone health are important in prostate cancer patients [193].

### 3.4. Immunotherapies and Their Combinations in Bone Metastatic Patients

Many clinical trials have evaluated immunotherapies as single agents or in combinations in patients with advanced cancers. However, only a very limited number of clinical trials have monitored and reported the effects on bone metastases, which we addressed in our recent publication [194]. Only about 1% (6/561) of publications with approved immunotherapies in breast, prostate, and lung cancer and melanoma patients reported results on bone metastases [194]. As an example, in the treatment of refractory metastatic CRPC patients treated with pembrolizumab in a phase 2 study, patients with bone predominant disease had better disease control rate and longer median overall survival than patients without bone metastases [69]. In patients with advanced or metastatic TNBC treated with atezolizumab and nab-paclitaxel, the combination treatment prolonged the median overall survival compared to patients treated with placebo and nab-paclitaxel [195]. Due to the limited number of eligible studies, no conclusions can be drawn about the efficacy. Clinical trials with immunotherapies specifying the effects on bone metastases are warranted, as they would allow to better understand metastasis-specific responses, improve patient selection in future clinical studies, and ultimately help in providing more effective treatments for patients with life-threatening bone metastases.

It should be recognized that while immunotherapies may have beneficial effects on bone metastasis, they may also have harmful effects on the skeleton that should be carefully monitored. Immunotherapies are associated with skeletal related adverse effects, including spinal cord compression, as well as fractures and lesions caused by increased bone resorption [196]. In a recent case report, nivolumab was associated with bone marrow necrosis [197]. Considering the interactions between immune and bone cells, it is likely that immunotherapies cause skeletal adverse effects, and the long-term adverse effects should be especially evaluated.

Additionally, in terms of both efficacy and safety, it is important to evaluate and understand the possible effects of preventive or concomitant treatments in heavily treated bone metastatic patients. For example, pre- and postmenopausal breast cancer patients have differential efficacy to zoledronic acid, which is linked to immunomodulatory effects of anti-estrogen treatment [160]. Similarly, prostate cancer patients are treated with many concomitant therapies, such as androgen deprivation therapy and glucocorticoids that also have immunomodulatory effects, and therefore, it becomes questionable whether combinations with immunotherapies show clinical benefit [198]. Importantly, bone-modifying agents are used in many clinical trials in combination with immunotherapies, though their use may not be equivalent and comparable. Therefore, considering the possible effects of bone-modifying agents on bone health, metastases, and study outcomes, there should be more awareness should of their use in future clinical trials.

## 4. Concluding Remarks

In this review, we introduce the novel concept of osteoimmuno-oncology (OIO), combining dynamic and multidirectional interactions between tumor, immune, and bone cells in bone metastasis. All of these fields are extensively studied individually, but they have not been combined earlier. We suggest that this concept should be the basis for developing novel immunotherapies against bone metastasis. Recently, the interest to treat bone metastatic cancer patients with immunotherapies has dramatically increased. Undoubtably, immunotherapies such as pembrolizumab, atezolizumab, nivolumab, durvalumab, and ipilimumab may provide potential treatment options for currently incurable bone metastases, and there are several other emerging targets and novel approaches to target immune cells in bone metastasis. Nevertheless, no clear clinical benefit has yet been demonstrated.

In order to develop novel therapies, and especially to enhance potential combination treatments, more preclinical and clinical research is urgently warranted to identify novel targets in bone metastasis. With this review, we would like to highlight the need for understanding the immune microenvironment in bone metastasis for better success in drug development. Bone metastatic tumor microenvironment is unique, and its immune cell milieu should be well characterized, also considering tumor heterogeneity and influences of concomitant treatments. A better understanding of OIO may finally reveal completely new opportunities for currently incurable bone metastases.

## Figures and Tables

**Table 1:** Summary of the current understanding of major anti- (−) and pro-tumoral (+) effects of immune cells on bone metastases, as per the published in vitro and in vivo studies and clinical findings. More detailed information is found in Section 2.1, Section 2.2, Section 2.3, Section 2.4, Section 2.5 and Section 2.6.

Immune Cell Type	Subtype	Effect on Bone Metastases (- Anti-Tumor Effect, + Pro-Tumoral Effect)	References
T cell	CD4+	(−) CD4+ cells have anti-tumor activity on primary and metastatic tumors (−) CD4+ cells prime survival of cytotoxic CD8+ cells (+) RANK/RANKL-mediated interactions with CD4+ and tumor cells increase invasion and dissemination of tumor cells and formation of metastases (+) TGF-β increases conversion of CD4+ cells to Tregs, which increases metastases	[68,71,72,73]
	CD8+	(−) CD8+ cells have anti-tumor activity on primary and metastatic tumors (−) CD8+ cells mediate immunological cell death (−) CD8+ cells remain in bone marrow several months after metastasis formation (−) CD8+ cells are associated with metastasis-free survival in patients (+) CD8+ cells increase migration, invasion, and expression of metastasis-associated genes (+) CD8+ cells have tumor-independent effects on bone cells	[26,68,74,75,76,77]
	Treg	(+) Tregs promote tumor cell homing to bone marrow (+) Tregs increase bone metastasis and cancer-induced bone resorption (+) Tregs are immunosuppressive and correlate with advanced stages and poor prognosis in patients	[26,78,79,80]
	NKT	(−) NKT cells can reject tumor cells in bone marrow (−) Low number or malfunction of NKT cells results in increased metastases	[26,81]
NK		(−) Loss of peripheral NK cells is associated with metastatic progression (−) Deletion of NK (genetic or activity) cells increases tumor growth and formation of metastases (−) NK cells can suppress metastases via IL-17, IL-28, IFN-γ and JAK signaling	[26,68,75,78,82,83,84,85,86,87,88,89,90]
MDSC		(+) MDSCs are associated with increased bone metastases and decreased survival (+) The number of MDSCs is highest in bone metastases (+) MDSCs contribute to the vicious cycle of bone metastasis (+) MDSCs in bone metastases can differentiate into osteoclasts (+) MDSCs increase metastasis via CCL5, CCL15, MMPs, and chemoattractants (+) MDSCs suppress proliferation and include the apoptosis of T cells and suppress the activity of NK cells (+) MDSCs activate Tregs	[60,68,73,78,82,91,92,93,94,95,96,97,98]
Macrophage	TAM/ M2	(+) TAMs cause tumor progression and the formation and growth of metastases (+) A high number of TAMs correlates with poor clinical outcome (+) TAMs secrete IL-10 and TGF-β, which decrease the activation of T cells (+) CD68+ and CD169+ macrophages increase growth of bone metastasis (+) CD169+ macrophages disable anti-tumor function of CD8+ T cells (+) Deletion of CD169+ macrophages inhibited cancer-induced new bone formation	[66,78,99,100,101,102,103,104,105,106,107]
DC	pDC	(+) DCs are increased in bone metastases (+) DC maturation is prevented in bone metastases (+) Bone marrow DCs deactivate CD8+ T cells via TGF-β (+) DCs recruit Tregs and MDSCs into the tumor	[68,78,83,108,109,110,111,112]
Neutrophil	TAN	(−) At early stages, neutrophils induce cytotoxic effects on cancer cells (+) Neutrophils participate in the formation of pre-metastatic niche (+) Neutrophils have immunosuppressive properties in metastases (+) N2 TANs are tumor-promoting, increasing angiogenesis, dissemination of tumor cells, and formation of bone metastases (+) N2 TANs secrete pro-metastatic CXCR4, VEGF, and MMP9	[78,113,114,115,116,117]

Abbreviations: CD: cluster of differentiation, NKT: natural killer T cell, MDSC: myeloid derived suppressor cell, TAM: tumor-associated macrophages, DC: dendritic cell, TAN: tumor-associated neutrophil. RANK: receptor activator of NFκB, RANKL: RANK ligand, TGF-β: transforming growth factor beta, IL: interleukin, IFN-γ: interferon gamma, JAK: janus kinase, CCL: CC chemokine ligand, MMP: matrix metalloproteinase, CXCR: CXC chemokine receptor, VEGF: vascular endothelial growth factor.
